# The Intellectual Profile of Adults with Specific Learning Disabilities

**DOI:** 10.3390/jintelligence11120223

**Published:** 2023-12-09

**Authors:** Eleonora Pizzigallo, Cesare Cornoldi, Serafino Buono, Santina Città, Francesco Viola, Enrico Toffalini

**Affiliations:** 1 Department of General Psychology, University of Padua, 35122 Padova, Italy; 2 Oasi Research Institute—IRCCS, 94018 Troina, Italy

**Keywords:** adults with SLD, intelligence, working memory, processing speed

## Abstract

Despite growing research on adults with specific learning disabilities (SLDs), evidence concerning their intellectual profile remains scarce. The present study examined the results of the administration of the Wechsler Adult Intelligence Scale—Fourth Edition to 301 adults diagnosed with SLDs and compared them to the results obtained from previous studies with a large sample of children with SLDs. The results showed that: (1) as observed among children, adults with SLDs also presented higher scores in the subtests implying reasoning (associated with the General Ability Index, GAI) and lower scores in the subtests involving working memory and processing speed; (2) the discrepancy between full-scale IQ and the GAI had a good predictive value in discriminating adults with and without SLDs; (3) the four-factor hierarchical structure of intelligence proposed for the general adult population held for adults with SLDs as well, even though there were substantial differences in the loadings and a five-factor structure could be more appropriate; (4) similarities as well as strong differences were present between adults and children with SLDs. In adults, scores on subtests were generally lower, particularly in working memory and processing speed. However, in some cases, scores were equal or even higher (as in the “Similarity” subtest) among adults, meaning that the discrepancy between the full scale and the GAI was accentuated.

## 1. Introduction

Specific learning disabilities (SLDs) are rooted in the biological profile of individuals and may persist across the lifespan, presenting various difficulties across ages (Swanson and Hsieh 2009). Even in adulthood, SLDs are listed as subgroups of neurodevelopmental disorders characterized by the presence of severe difficulties in reading and/or writing and/or mathematics not due to sociocultural, emotional, general intellectual, and neurological problems (cf. the Diagnostic and Statistical Manual of Mental Disorders, 5th edition (DSM-5), APA (2013); and the International Classification of Diseases, 10th Revision (ICD-10), WHO (1993)), and are safeguarded by health systems (as for Italy, ISS 2011; SNLG ISS 2022).

According to the DSM-5, the prevalence rates of SLDs range internationally between 3 and 5% (APA 2013). Other studies report higher prevalence (up to 5–17% of the population worldwide) depending on the definition and assessment methods used (cf.  Gerber 2012) and on their actual identification. A negative prognosis in terms of working life is reported, with higher rates of frustration and unemployment (Bynner and Parsons 2006; Daniel et al. 2006). Thus, it is important to expand awareness of SLDs in late adolescence and adult ages.

### 1.1. Cognitive Weaknesses of Adults with SLD

So far, research on adults with SLDs has focused mainly on specific aspects that are critical in children with SLDs, without particular consideration for specificities that could characterize adulthood. Various weaknesses in cognitive abilities associated with SLDs have been identified in verbal short-term memory, attention (sustained attention, inhibiting responses in the presence of distracting stimuli), visual-spatial processing (visual-spatial perception and manipulation tasks), executive functioning (planning, organizing, and behavior monitoring), sequential (spatial underspecified word form representation) and processing speed (Alloway and Alloway 2010; Callens et al. 2014; Kinsbourne et al. 1991; Peter et al. 2021; Reis et al. 2020; Trecy et al. 2013). These findings have suggested that SLDs are associated with a range of underlying cognitive difficulties.

Most studies have been concerned with reading and spelling disorders. It has been confirmed that individuals with dyslexia commonly experience difficulties with spelling as well (Hanley 1997; Kemp et al. 2009; Maughan et al. 2009) and exhibit deficits in various cognitive processes, which may contribute to their difficulties in writing, producing texts with more grammatical errors, poorer cohesion, and lower syntactic complexity (Bogdanowicz et al. 2014). Furthermore, adults with dyslexia may show deficits in several cognitive domains, including phonological processing (difficulty with phoneme awareness, phonological memory, and rapid naming tasks). For example, Snowling et al. (1997) found that university students with dyslexia present specific weaknesses in phonological processing tasks, such as non-word reading, spoonerism accuracy and speed, phonemic fluency, and phoneme deletion. Hatcher et al. (2002) found that dyslexia is associated with weaknesses in phonological processing, verbal short-term memory, and rapid automatized naming. Their discriminant function analyses indicated that dyslexia in adulthood can be confirmed with 95% accuracy using four tests: spelling, non-word reading, digit span, and writing speed. Additionally, Re et al. (2011) found that the ability to write in a condition of articulatory suppression has a high discriminative capacity.

A few studies have considered the case of mathematical disabilities (see, e.g., Vigna et al. 2022). In general, it was found that similar cognitive weaknesses can be found in adults with either dyslexia, dyscalculia, or both. For example, Cornoldi et al. (2022) revealed that adults with dyslexia present difficulties in reading and writing numbers comparable in severity to those exhibited in reading and writing words. Wilson et al. (2015) found that issues with phonological processing, rapid naming, and verbal short-term memory are associated with both dyslexia and math disabilities.

Therefore, important evidence shows that not only tests of reading, writing, and mathematics but also cognitive tests help detect and characterize adults with SLDs. Nergård-Nilssen and Hulme (2014) observed that a combination of tests, including cognitive functioning measures, predicted dyslexia in a sample of Norwegian adults with good accuracy. However, research is mainly focused on specific aspects of cognitive functioning without any reference to a framework of general intellectual functioning. General intelligence (as traditionally described by an IQ measure) is often used as a control measure to exclude the possibility of overall borderline functioning or disability. On this basis, it is unsurprising that adults with SLDs tend to exhibit fairly good intellectual abilities. Hatcher et al. (2002) discovered that students with dyslexia demonstrate lower performance on all cognitive tasks except for two tests that measure verbal reasoning, derived from the Wechsler Scales, and non-verbal reasoning (Raven 1938). Additionally, in a meta-analysis (Swanson and Hsieh 2009), effect sizes greater than 0.60 have been found for verbal memory, RAN, and vocabulary but not for general intelligence and problem solving.

### 1.2. Intellectual Characteristics of Individuals with SLD

Despite an average level of intelligence, individuals may present strengths and weaknesses within specific areas of intellectual functioning. Research on children with SLDs has emphasized that differentiating various aspects of intelligence and finding strengths and weaknesses may offer important information on their profiles. Thus, it confirms the importance of conducting a multi-componential assessment of intelligence, at least in individuals with SLDs. This approach, which has been largely criticized, especially by researchers favoring a g-centric approach (e.g., Beaujean 2017), has found empirical and clinical support (e.g., Toffalini et al. 2017a, 2017b). This research has mainly used the Wechsler Intelligence Scales for Children (WISC), the largest intelligence battery used globally, which seems particularly appropriate for identifying the characteristics of individuals with neurodevelopmental disorders. Research using the WISC-IV (Wechsler 2004) has revealed that children with SLDs exhibit a four-factor structure of intelligence, comparable to the one of typically developing (TD) children (Cornoldi et al. 2014; Giofrè and Cornoldi 2015; Toffalini et al. 2017b). However, there are notable differences in the loadings of certain factors. Children with SLDs have lower loadings on *g* for the verbal comprehension (VC) factor compared to the perceptual reasoning (PR) factor and lower loadings for the two factors concerning working memory (WM) and processing speed (PS). Additionally, children with SLDs obtain average scores similar to those of TD children in the two reasoning indexes (VC and PR) associated with the General Ability Index (GAI) but lower scores in the two indexes representing cognitive competency, associated with working memory and processing speed. The discrepancy between GAI and full-scale IQ can be valuable in identifying cases of learning disorders. This is consistent with the fact that children with SLDs have difficulties in both verbal working memory and processing speed.

It remains unclear whether similar results can be replicated with adults using the adult version of the Wechsler Scale, i.e., the Wechsler Adult Intelligence Scale, 4th version (WAIS-IV). The WAIS-IV shares the four main indexes and a substantial portion of the subtests with the WISC-IV, which is typically used until the age of 16 years (Wechsler 2004). Afterwards, the WAIS-IV is employed throughout the rest of the lifespan.

Evidence concerning the continuity between the intellectual characteristics of children and adults with SLDs is scarce. Rack (1997) identified continuity in the cognitive profile of people with SLDs along their lifespan. However, Laasonen et al. (2009), using the previous version of the WAIS, i.e., the WAIS-III, with a group of more than 100 adults with dyslexia, found that all adults shared difficulty in processing speed, similar to the one observed with children, but specific issues with perceptual or memory abilities were found only in specific subgroups. Similar, but not identical, difficulties have been observed by Godoy de Oliveira et al. (2014), who studied 31 Brazilian adults with dyslexia. These adults exhibited poorer performance in Processing Speed and Working Memory subtests, but also underperformed on Verbal IQ and Picture Completion, Matrix Reasoning, Similarities, and Vocabulary subtests, with differences that were particularly relevant in Coding.

The cognitive profile of individuals with SLDs may change also due to the experiential limitations related to their reading, writing, and mathematical difficulties, as suggested by Gunnel Ingesson’s longitudinal study (2006), which compared the intellectual characteristics of 65 subjects with dyslexia when they were 12 years old and after an average interval of six and a half years. The study revealed a significant relative decrease in verbal IQ, along with a complementary increase in non-verbal IQ. Furthermore, a study with gifted individuals with SLDs (Toffalini et al. 2017c) suggested that over time they might compensate for the processing difficulties, which offer a greater opportunity to use visual strategies and control, like the symbol search subtest, but show an increased difficulty, compared to that of their controls, in the digit span subtest.

These differences could also be emphasized by the fact that children and adults with SLDs do not necessarily represent the same population, as not all children with SLDs maintain the diagnosis when they become adults, while some individuals receive a diagnosis of an SLD in their adulthood (e.g., the National Italian Consensus Conferences on Specific Learning Disorders; SNLG ISS 2022). In sum, there is a need for a deeper understanding of the intellectual characteristics of adults with SLDs.

### 1.3. The Present Study

In the present study, we examined a large sample of adult individuals with a diagnosis of an SLD who were administered the ten basic subtests of the WAIS-IV. We decided to use the WAIS-IV as this was the only test used by all the centers involved in the study and because it offered the possibility of directly comparing adults and children with SLDs. Furthermore, the WAIS-IV represents one of the tests for adults with the most robust psychometric properties (see Orsini and Pezzuti 2013, 2015), despite the fact that some relevant evidence concerning its use in SLDs is missing (e.g., factorial invariance, factorial structure, discriminative power). In this regard, one of the aims of the present study was to fill this gap.

We focused on four main research questions:

First, we looked at the average intellectual profile of adults with a diagnosis of an SLD. Based on the previous literature on children and adults, we predicted substantial disparities between the highest mean scores concerning the subtests involving non-verbal and especially verbal reasoning, and the lowest scores concerning working memory and especially processing speed. An associated issue concerns the possibility offered by the WAIS-IV of considering more general aspects of intelligence rather than the performance at the single subtests of the battery. In fact, the authors of the WAIS-IV assume that four factors and corresponding indexes may be derived by the administration of the WAIS. These include verbal comprehension (VCI) concerning verbal reasoning, perceptual reasoning (PRI) concerning visuospatial and fluid reasoning, working memory (WMI) concerning the ability to temporarily hold information in memory and manipulate it, and processing speed (PSI) concerning the ability to process visual information quickly, maintaining the attention on the task for short durations. Grouping the intelligence scores into these four indexes proved to be highly informative in the case of children (e.g., Toffalini et al. 2017b), while evidence of the corresponding four WAIS-IV indexes remains limited. An exception is constituted by a preliminary study of D’Elia et al. (2023) that involved 10 Italian university students, which found that they were poor in WM and PS but better in VC and PR.

A second issue examined in our study was whether the intellectual profile can be used for discriminating between individuals with and without SLDs, even in the absence of academic measures. This possibility was denied by Maller and McDermott (1997), who, notably, employed a previous version of the WAIS (WAIS-R). It is possible that the indexes offered by the WAIS-IV, designed to be analogous to the WISC-IV (Wechsler 2004), have better discriminative power. A study comparing the profiles of children with SLDs vs. those of the TD population showed that the diagnostic power of a combination of the four indexes was reasonably good, AUC = 0.78 (0.76, 0.79) (Giofrè et al. 2017). Therefore, the intellectual profile could provide important information in uncertain cases where, due to a series of problems (e.g., foreign origin of the individual, language difficulties, etc.), measures concerning academic performance are not reliable. The information could be useful also in cases of suspected malingering, as individuals who want to exhibit unpossessed weaknesses would offer false performances in a generic way, not coherent with the actual specific observed pattern.

The third issue concerned whether the factor structure of intelligence proposed by the authors of the WISC-IV and WAIS-IV, distinguishing between four main factors, also holds true for adults with SLDs or presents specificities. Research using a previous version of the WAIS, i.e., the Swedish version of WAIS-R, on 88 adults with dyslexia, conducted by Alm and Kaufman (2002), revealed a three-factor structure with a verbal comprehension factor, a perceptual organization factor, and a freedom from distractibility factor, the latter representing the weakest component in SLDs. The latest version of WAIS-IV features a four-factor structure that seems more appropriate for describing intellectual profiles.

Giofrè and Cornoldi (2015) found that a four-factor configuration was adequate for children with SLDs, although with substantial differences in the structure of loadings vis-à-vis the TD population. The WM and PS factors presented much weaker loadings on the g-factor in children with SLDs vis-à-vis TD. Differences in the factorial structure of intelligence between adults and children with SLDs may stem not only from age disparities but also due to variations in specific subtests between the WAIS-IV and WISC-IV. A major difference concerns the second WM subtest of the WAIS-IV, which requires arithmetic reasoning and calculations, whereas in the case of the WISC-IV, it concerns the mental remembering and reordering of letters and numbers. It should be noted that the arithmetic subtest presents a specific challenge for individuals with SLDs, who often struggle with numbers. However, it also requires reasoning operations, which partly involve entailing WM processes while largely entailing fluid reasoning operations, in line with the new proposals based on the CHC model (McGrew 2009). As suggested by research on the intelligence structure emerging from the latest WISC version (Wechsler 2014) and considering a factor for fluid reasoning, a five-factor structure might be even more appropriate than a four-factor structure.

The fourth issue directly examined differences between the intellectual profiles of adults with SLDs and those of children with SLDs, with a focus on the subtests that are nearly identical in the WISC-IV and the WAIS-IV. In a subgroup of adults, data on supplementary subtests of the WAIS-IV were available, so we could run a direct comparison with all WISC-IV subtests for at least a part of the sample. Based on previous literature (e.g., Callens et al. 2014), we predicted that the discrepancy between reasoning and processual factors, already evident in children with SLDs (e.g., Cornoldi et al. 2014), could be even more emphasized in adults, resulting in increased scores for reasoning and decreased scores concerning working memory (see Toffalini et al. 2017c).

## 2. Materials and Methods

### 2.1. Participants

A total of 301 adult cases previously diagnosed with SLDs were examined. They were aged between 16 and 56 years (*M_age_* = 21.34; *SD* = 7.02; 50.8% females) and located in four Italian regions (Veneto, Emilia-Romagna, Liguria, and Sicilia). The cases were diagnosed by expert licensed psychologists in clinical centers. The diagnoses were established according to the ICD-10 (WHO 1993), the DSM-5 (APA 2013), and the National Italian Consensus Conferences on Specific Learning Disorders (ISS 2011; SNLG ISS 2022). The individuals’ difficulties could not be attributed to socioeconomic or educational disadvantage, sensory, neurological, or intellectual deficits (APA 2013). They were all native Italians and did not present any other neurodevelopmental disorders. The assessment was conducted using a nationally standardized set of tests, designed to measure achievement in different academic fields.

The Italian standardized measures, including the DDE-2 battery, a collection of MT batteries, and the LSC-SUA battery, collectively assess a wide range of learning abilities, such as reading, writing, and calculation skills, with each battery specifically designed to assess abilities relevant to the individual’s age and scholastic grade (e.g., Montesano et al. 2020; Sartori et al. 2007). In order to receive a diagnosis of an SLD in accordance with the guidelines outlined in Italian legislation (Law 170/2010 2010), it is necessary to observe standardized test scores in reading accuracy falling below the 5th percentile or 2 standard deviations (*SD*) below the age mean or grade mean in reading, writing, and/or mathematical achievement tests. A less stringent criterion (1.5 *SD*s) is applied if additional information indicates that a problem is persistent and has severe adaptive implications for the individual’s everyday life.

Ethical approval from an Institutional Review Board was not required as the research involved a secondary analysis of archival data originally collected for clinical assessment by the centers collaborating with the study; the data were fully anonymized for the researchers.

Based on the classification adopted in Italy and following the ICD-10, the distribution of the diagnosis was: 135 cases of specific reading disorders (F81.0), 7 cases of specific spelling disorders (F81.1), 15 cases of a specific disorder of arithmetical skills (F81.2), 132 cases of a mixed disorder of scholastic skills (F81.3), and 10 cases of other developmental disorders of scholastic skills (F81.8).

A large sample of 1625 children diagnosed with SLDs, aged between 7 and 17 years (*M_age_* = 11.56, *SD* = 2.44; 39.2% females), was used for comparison. The data from this sample had already been described and analyzed in a set of previous articles (Giofrè et al. 2017, 2019; Toffalini et al. 2017a), and here are presented only as a benchmark for corresponding results on adults. These developmental cases were diagnosed using the same psychometric criteria required by the national guidelines as detailed above. The distribution of cases was as follows: 467 with specific reading disorders, 198 with specific spelling disorders, 122 with a specific disorder of arithmetical skills, 622 with a mixed disorder of scholastic skills, and 216 with other disorders of scholastic skills.

### 2.2. Instrument

All participants were assessed with the Italian adaptation (Orsini and Pezzuti 2013, 2015) of the fourth version of Wechsler Batteries for Adults (Wechsler 2008). The WAIS-IV assesses intelligence in individuals aged between 16 and 90 years. The Italian version was first standardized with a sample of 1424 adults (697 males and 727 females), divided into 9 age groups. Age ranged between 16:0 and 69:11 (*M* = 36.4; *SD* = 9.8). The Italian adaptation demonstrates strong internal consistencies, excellent test-retest and inter-rater stability, and standard errors of measurement comparable to those of the American normative population. Detailed data can be found in the Manual (Orsini and Pezzuti 2013).

The whole sample was administered the ten basic subtests (standardized scores: *M* = 10; *SD* = 3). These basic subtests are, in order of administration: Block Design (BD; requiring the reconstruction of visual patterns by appropriately combining faces of a series of cubes), Similarities (SI; requiring indication of an aspect shared by two concepts), Digit Span (DS; requiring immediate recall and repetition of digit series, progressively increasing in length, both in ascending and descending orders), Matrix Reasoning (MR; requiring the selection of a figure from several options to complete an array of pictures), Vocabulary (VC; knowledge and definition of words’ meaning), Arithmetic Reasoning (AR; requiring mental calculation of various mathematical problems orally explained), Symbol Search (SS; requiring participants to swiftly determine whether a specific target stimulus is present within a group of symbols), Visual Puzzles (VP; requiring the reconstruction of a figure by combining pieces), Information (IN; requiring answers to a series of general encyclopedic-type questions, adapted to Italian culture), and Coding (CD; requiring rapid association of numbers to symbols within a grid).

In some cases, five supplementary (optional) subtests were also administered for an in-depth analysis of the cognitive process (or, occasionally, to replace the invalid core subtests). These supplementary subtests were administered according to the order proposed by the original protocol, and included Letter-Number Sequencing (LN; requiring participants to re-order and remember disarranged sequences of letters and numbers), Figure Weights (FW; requiring the selection of different weights’ images to maintain balance on a scale), Comprehension (CO; requiring the understanding and ability to answer questions concerning life problems), Cancellation (CA; requiring rapid identification of a target stimulus among several distractors), and Picture Completion (PC; requiring identification of a missing detail from an image).

In addition to the FSIQ, the WAIS-IV allows computing four main indexes (standardized scores *M* = 100; *SD* = 15) that represent the hypothesized four-factor structure of intelligence. Verbal Comprehension (VCI) is based on the scores of the SI, VC, and IN subtests, Perceptual Reasoning (PRI) is based on the scores of the BD, MR, and VP subtests, Working Memory (WMI) is based on the scores of the DS and AR subtests, and Processing Speed (PSI) is based on the SS and CD subtests. The battery also offers the opportunity to calculate two additional indexes, the General Ability Index (GAI) and the Cognitive Proficiency Index (CPI). The GAI is a composite score, sometimes considered an alternative general estimate of intelligence, based on the subtests contained in the Verbal Comprehension Index and Perceptual Reasoning Index. The CPI, on the other hand, is composed of Working Memory and Processing Speed scores and concerns the use of learning processes.

### 2.3. Data Analysis

All analyses were computed using R software with the “lme4” (Bates et al. 2015), “lavaan” (Rosseel 2012), and “pROC” (Robin et al. 2011) packages.

Descriptive statistics of the mean standardized scores for the main subtests and WAIS-IV indexes were computed, and their correlations with age were observed. The mean profile of scores at the indexes was examined using a mixed-effects linear model, with the standardized scores as the response variable, the index as the fixed effect, and individuals as random effects (with random intercepts). Subsequently, the four indexes of the WAIS-IV were used to discriminate between adults with SLDs and adults with TD using logistic regression with indexes as predictors and status (SLD vs. TD) as the dichotomous dependent variable (as in Giofrè et al. 2017). As a measure of discriminatory power, the area under the curve (AUC) of the receiver operating characteristic (ROC) curve was calculated, with an AUC = 0.50 representing no discriminatory power. The TD population was simulated using normative data and the correlation matrix provided by the Italian standardization of the battery, as in Giofrè et al. (2017).

A confirmatory factor analysis (CFA) was adopted to examine the structure of intelligence, following previous analyses (Giofrè and Cornoldi 2015). Two alternative models were fitted. First, a hierarchical four-factor structure of the WAIS-IV (with a higher-order g-factor at the top and four first-order factors as latent variables) and then a hierarchical five-factor structure (including a new factor representing Fluid Reasoning) were investigated. The sets of parameters considered were loadings (standardized coefficients), their 95% confidence intervals (CIs), and the residual variances. The four-factor CFA model had 34 parameters and was estimated using the full-information maximum likelihood (FIML) method to exploit all available data. For goodness of fit, the following fit indexes suggested by Jöreskog and Sörbom (1993) were used: the chi-square (χ^2^), root mean square error of approximation (REMSEA), standardized root mean square residual (SRMR), the comparative fit index (CFI), the non-normed fit index (NNFI), and the Bayesian Information Criterion (BIC) was used for model comparisons.

Finally, to compare adults and children with SLDs, the mean weighted scores for all subtests of the WAIS-IV and WISC-IV were computed. Additionally, average profiles of scores (with their 95% CIs) at the indexes, estimated using a mixed-effects linear model, were tested in both adult and children samples using the same analysis as previously described.

## 3. Results

### 3.1. Preliminary Analysis and Descriptive Statistics

Table 1 presents the average standardized scores for the whole sample of participants with SLDs. The number of cases reported in the second column varies as valid available information varied according to the subtest. For corresponding descriptive statistics on the developmental sample, see Supplemental materials, Table S1. Spearman’s rank correlation coefficients between age and weighted scores were all small, ranging between 0.16 (Similarities) and −0.12 (Figure Weights). The only significant correlations (with *p* < 0.05) concerned two verbal subtests: Similarities, r = 0.16, *p* = 0.007, and Vocabulary, r = 0.13. For this reason, subsequent analyses were carried out with all participants in a single analysis.

As the mean weighted score for the general population in any subtest is defined as 10 (*SD* = 3), a comparison can be made by examining to what extent the mean scores of the adults with SLDs deviate from 10. SLD cases showed below-average performance in Digit Span and Arithmetic, composing the Working Memory Index, as well as in Coding and Symbol Search, composing the Processing Speed Index. Mean scores in Verbal Comprehension and Perceptual Reasoning areas were around the population average.

Concerning the Working Memory Index, Arithmetic Reasoning is a subtest that might cause additional difficulties for a share of adults with SLDs due to their weakness in the arithmetic area (Cornoldi et al. 2022).

### 3.2. Diagnostic Power of Indexes

Following the methodology employed by Giofrè et al. (2017) in studying the child population, we proceeded to assess the diagnostic capability of the WAIS-IV standardized indexes in distinguishing individuals with SLDs from those with TD. The data of TD individuals were simulated based on normative scores and their correlation matrix reported in the WAIS-IV standardization manual. Subsequently, logistic regression was computed. All four index scores significantly concurred in predicting whether an individual belonged to the SLD or the TD group: VCI: odds ratio (OR) = 0.96, *p* < 0.001; PRI: OR = 0.95, *p* < 0.001; WMI: OR = 1.11, *p* < 0.001; PSI: OR = 1.04, *p* < 0.001. The resulting AUC (area under the curve) was 0.83 (95%CI: 0.80, 0.86), which represented good discriminatory power, even higher than that observed with the developmental population, where the AUC was 0.78 (0.76, 0.79).

### 3.3. Confirmatory Factor Analysis

**Figure 1:**
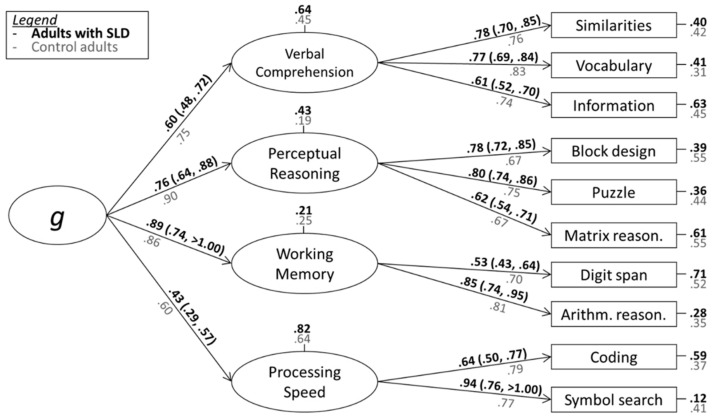
Results of the confirmatory factor analysis (standardized loadings and 95% CIs) for adults with SLDs (black parameters in bold) and the population without SLDs (grayparameters) based on a four-factor structure.

Figure 1 presents the results of the confirmatory factor analysis (CFA) with the four factors modeled as latent variables, and a superordinate g factor, calculated for the 301 observations with complete information on all 10 basic subtests. The results were compared with the results obtained with 1424 adults of the standardization sample and reported in the Italian Manual (Orsini and Pezzuti 2013). The standardized fit indexes were acceptable, χ^2^(31) = 78.63, *p* < 0.001, RMSEA = 0.07, SRMR = 0.05, CFI = 0.95, NNFI = 0.92, and BIC = 14,140.632, suggesting that it is appropriate to also consider the performance of adults with SLDs based on the four-factor structure of the WAIS-IV battery.

The loadings identified in adults with SLDs differed from the pattern observed in the control group. For SLDs, the loadings were overall lower (except for WMI). The discrepancies in the loadings of g on the WMI and PRI could stem from the inclusion of Arithmetic Reasoning in the WMI factor. This particular subtest predominantly loads on fluid reasoning, as demonstrated in its repositioning in the WISC-V (Wechsler 2014). Thus, in an alternative CFA, we re-examined the intelligence structure using the WISC-V five-factor structure.

In the alternative CHC-inspired model, Matrix Reasoning and Arithmetic Reasoning subtests were combined into a factor representing Fluid Reasoning. As a result, the Working Memory Index remained represented by a single subtest (Digit Span). To prevent the risk of a factor becoming isomorphic with an observed indicator, the standardized loading of Digit Span on WM was fixed to 0.80, which represents an arbitrary but plausible value. Additionally, the residual variance for the Fluid Reasoning factor was estimated to be negative due to above 1.00 standardized loading on g. To address this problem, the residual variance was set to zero.

**Figure 2:**
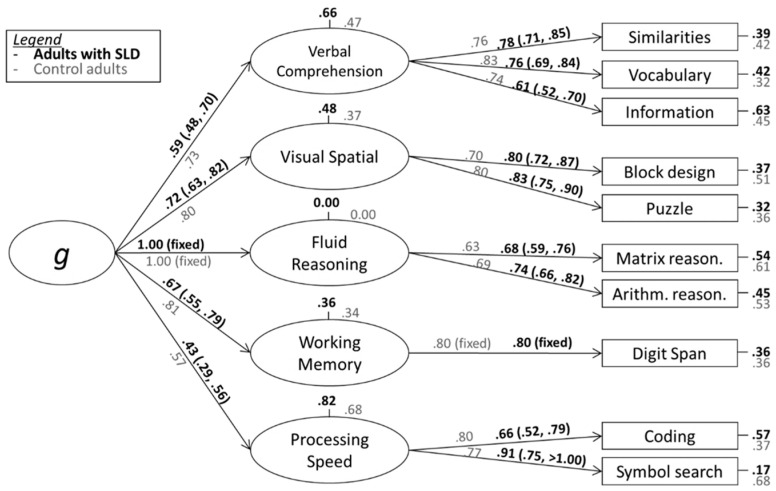
Results of the confirmatory factor analysis (standardized loadings and 95% CIs) for adults with SLDs (black parameters in bold) and the population without SLDs (gray parameters) based on a five-factor structure.

Figure 2 shows the hierarchical five-factor structure of the WAIS-IV with standardized coefficients for the samples of adults with SLDs and the control group. The five-factor structure had acceptable fit indexes χ^2^(31) = 69.14, *p* < 0.001, RMSEA = 0.06, SRMR = 0.04, CFI = 0.96, and NNFI = 0.94, which were slightly better than in the case of the four-factor structure, as indicated by BIC = 14,125.444, confirming the overall adequacy of the model with a five-factor structure.

As seen in Figure 2, once again the loadings were lower in SLDs than they were in the control TD population. Focusing on the standardized loadings of Verbal Comprehension, Visual Spatial, and Perceptual Reasoning Indexes (λ = 0.59, λ = 0.72, and 0.43, respectively), they were almost the same as in the previous model. On the contrary, the loading of the WM factor was much lower than in the four-factor structure (λ = 0.67 in the five-factor model vs. 0.89 in the four-factor structure), confirming that the previous high loading on g of WMI was due to the inclusion of the Arithmetic Reasoning subtest in WMI. The test manual lacks a straightforward method for calculating the index scores based on this grouping of the subtests and does not seem particularly appropriate due to the specificities of the Arithmetic subtest in SLDs. Nevertheless, considering that the standardized scores of the SLD group were 9.99 and 7.34, respectively, on the Matrix and Arithmetic Reasoning subtests, we anticipated a relatively low mean score for SLDs in Fluid Reasoning.

As the scores in both the Arithmetic Reasoning and the Information subtests might be directly affected by school achievement, thus being potentially biased in individuals with SLDs, we re-ran both models after removing these two subtests. The results are presented in Supplemental Materials, Figures S1 and S2. Overall, the patterns of coefficients were similar, but with a major change in the loading of g to WM in the four-factor model on adults with SLDs, which dropped from 0.89 to 0.56 (a smaller variation, from 0.67 to 0.57, was observed in the five-factor model). This is consistent with the notion that working memory is mostly independent from the g factor in individuals with SLDs, as already noted in children (Giofrè and Cornoldi 2015).

### 3.4. Comparison with Children with SLD

**Figure 3:**
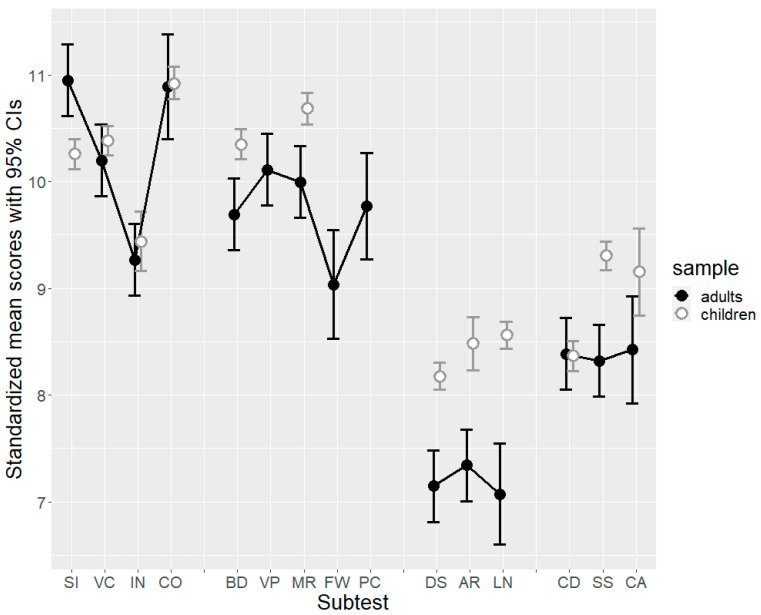
Mean standardized scores of subtests (with 95% confidence intervals). SI = Similarities; VC = Vocabulary; IN = Information; CO = Comprehension; BD = Block Design; VP = Visual Puzzles; MR = Matrix Reasoning; FW = Figure Weights; PC = Picture Completion; DS = Digit Span; AR = Arithmetic Reasoning; LN = Letter-Number Sequencing; CD = Coding; SS = Symbol Search; CA = Cancellation.

The final analysis, based on the availability of data from the developmental sample (see Participants section), compared the weighted scores of children and adults with SLDs in the Wechsler Scales subtests. This analysis provided a unique opportunity of cross-comparison, specifically within the similar subtests of WISC-IV and WAIS-IV, among individuals that are facing the same clinical condition. The results of the analysis are synthesized in Figure 3 (notice that in the case of adults, the confidence intervals were larger, as they had been derived from a smaller sample). These scores were gathered from the complete sample across the 10 core subtests, and for some participants, the data were available for one or more supplementary subtests.

Children and adults were similar in many ways, with a better performance on the subtests associated with VCI and PRI, and a worse performance on the subtests associated with WMI and PSI. However, in some cases, there were substantial differences, as confirmed by non-overlapping 95%CIs. In particular, in a specific subtest of VCI, Similarities, the group of adults (*M* = 10.95) exhibited a better performance compared to the group of children (*M* = 10.26). Their performance in the remaining subtests remained consistent, indicating a general preservation of verbal abilities among adults. However, adults’ scores were lower than those of children in two PRI subtests (Block Design and Matrix Reasoning), all WMI subtests, and two PSI subtests (Symbol Search and Cancellation). Coding was the only PSI subtest in which adults (*M* = 8.39) and children (*M* = 8.37) obtained similar results. The discrepancy observed among children between Coding and Symbol Search disappeared in adults.

In conclusion, the average profiles of the four indexes for both groups, calculated based on the ten basic subtests and estimated using a mixed-effects linear model, were examined (Table 2). The model, applied to standardized scores from the main indexes of WAIS-IV and WISC-IV, revealed elevated scores in VCI and PRI, while indicating weaknesses in WMI and PSI. The β coefficients estimate the mean differences between each of the four factors and the intercept (i.e., the FSIQ). They reveal that, among adults with SLD, mean scores significantly varied in the following order: VCI > PRI > PSI > WMI. The average profile of adults with SLDs is broadly similar to that of children, although lower on average (cf. the FSIQ means). Interestingly, one notable difference in the profile concerned the index with the highest mean score: for children, it was PRI, whereas for adults it was VCI, with a slightly lower average IRP. Instead, the WM area was particularly low in both adult and children samples.

## 4. Discussion

The case of adults with SLDs has been receiving increasing interest due to the adoption of a lifespan perspective, which considers neurodevelopmental disorders throughout individuals’ lives. Although certain characteristics of adults with SLDs have been outlined in the existing literature, a comprehensive examination of the intellectual profile of this population has been lacking; the available evidence in this regard has not been sufficiently robust.

In the present paper, we addressed four main research questions. First, we examined the intellectual profile of adults with SLDs, using the information collected through the administration of the ten basic subtests of the WAIS-IV. It has been found that adults with SLDs attained a mean score close to the normative average in verbal and non-verbal reasoning subtests. However, they achieved substantially lower scores in subtests involving working memory and processing speed. These results confirm previous evidence that claimed that adults with SLDs have good reasoning abilities (e.g., Hatcher et al. 2002) and difficulties in phonological short-term memory (e.g., Callens et al. 2014; Hatcher et al. 2002; Swanson and Hsieh 2009) and processing speed (e.g., Laasonen et al. 2009). Nevertheless, in contrast to findings reported in prior studies (Godoy de Oliveira et al. 2014; Gunnel Ingesson 2006), there was no decrease in verbal abilities in adults vis-à-vis children. This difference could be attributed to distinct sample characteristics, including a limited sample size in one instance, variations in participant ages (with one study exclusively involving late adolescents), and disparities in the administered tasks.

Regarding the WAIS-IV, our findings align with observations made by D’Elia et al. (2023), despite their study being conducted with a limited number of subjects. It should be noticed that other evidence (e.g., Godoy de Oliveira et al. 2014) has suggested that the Coding subtest, which required participants to handwrite and process numerical information, may be particularly critical among adults with SLDs. Nevertheless, our results show that the same effort can be encountered in another processing speed subtest, such as Symbol Search, which requires participants to process only visual materials. In addition to that, adults with SLDs performed poorly in the other working memory subtest. The performance of the adults with SLDs was poor, but the result could partly be due to their extensive mathematical difficulties, although in some cases, their diagnosis does not specifically refer to mathematics (Cornoldi et al. 2022).

A second research question pertained to whether the intellectual profile could be used per se for discriminating between individuals with and without SLDs. This possibility was denied by Maller and McDermott (1997), who, notably, employed a previous version of the WAIS (WAIS-R) that did not distinguish the four factors and did not measure the corresponding indexes. A prior study that compared profiles emerging from children with SLDs with those of the TD population, calculated the discriminative power of the four indexes combined and demonstrated reasonably good diagnostic power, AUC = 0.78 (0.76, 0.79) (Giofrè et al. 2017). In the present study, we adopted the same methodology and found an even higher AUC of 0.83 (0.80, 0.86). As a result, it has been demonstrated that it was possible to discriminate, with good approximation, individuals with and without learning difficulties, even without the administration of learning tests.

The above-described results have important implications for the diagnosis, especially in cases where measures of reading, writing, and arithmetic are considered unreliable (e.g., due to poor schooling or cultural-linguistic differences that can also affect cognitive abilities). They can also aid in differentiating between individuals with genuine disorders and those feigning symptoms, in conjunction with factors previously identified by other researchers (e.g., Re et al. 2011; van den Boer et al. 2018). However, it is important to note that comparable discriminative power is evident only when individuals with SLDs are compared to those without any neurodevelopmental disorders, whereas evidence is needed for more subtle differentiations within neurodevelopmental disorders.

The third research question examined the extent to which the four-factor structure of intelligence, as proposed by the authors of the WISC-IV and WAIS-IV, applies to adults with SLDs and whether it exhibits specific characteristics. In the context of the WISC-IV, Giofrè and Cornoldi (2015) determined that the four-factor structure was acceptable for children with SLDs. However, notable disparities emerged when compared to typically developing (TD) children, particularly in the WM and PS factors, and to some extent in VC. These factors were less heavily loaded by a general factor in the case of SLDs compared to those in the TD population. The current study yielded comparable results, showing that a four-factor structure had a good fit and suggesting that the three-factor structure proposed by Alm and Kaufman (2002) aligns with the characteristics of the earlier version of the WAIS they employed.

When comparing the structure obtained from adults with SLDs to that of the control group, however, we found that the *g* factor did not load on the working memory factor substantially less than it did in the control population. This seems to mark a difference compared to what was previously reported on children (e.g., Giofrè and Cornoldi 2015).

The four-factor structure, however, seems to present limitations that are particularly evident in the case of adults with SLDs, as some subtests, like Information and especially Arithmetic, might reflect specific difficulties directly related to SLDs rather than general aspects of cognitive functioning. Arithmetic reasoning is factorially complex because it involves the working memory, but it may also involve fluid reasoning (as fully acknowledged in the WISC-V model), and partly reflect school achievement, making it inadequate to measure any factor in individuals with SLDs. A tentative factor analysis based on a five-factor structure, as suggested by Wechsler (2014), appeared to provide a more satisfactory fit than the four-factor structure and showed that the fifth factor, concerning fluid intelligence, was highly loaded by *g*. In additional analyses (Supplemental Materials, Figures S1 and S2), Arithmetic reasoning was removed from the working memory factor. The results from this set of analyses suggested that the loading of *g* on working memory was much lower than shown in the original model, and more in line with what was previously observed in children (e.g., Giofrè and Cornoldi 2015). Overall, results suggested that the *g* factor generally presented lower loadings, thus accounting for a lower share of variance of the first-order factor in the population with SLDs vis-à-vis the general population. This is especially evident in verbal comprehension, processing speed, and most likely working memory (as discussed above). A direct implication of this is that considering the individual profile of factor scores might be justified and more informative than just considering full-scale IQ in this population vis-à-vis the general population. This was also corroborated by the good diagnostic power shown by the profile of scores in discriminating between cases and controls. Similar suggestions have already been made for the child population with SLDs (e.g., Giofrè et al. 2019).

The fourth research question aimed to explore potential disparities in the average intellectual profiles between adults with SLDs and children with SLDs. The comparison was carried out using data from a previous study program, specifically drawing upon observations gathered from 1049 children diagnosed with an SLD. The four-factor model used to create the WISC and WAIS batteries features the same latent factors, and as a consequence, most subtests (including supplementary ones) are nearly the same (except for partly different materials) and are therefore comparable. This comparison offered important suggestions. The first general observation was that the average profiles of both adults and children with SLDs were largely overlapping, providing preliminary evidence that suggests continuity across the lifespan. Longitudinal studies are needed to better determine the actual degree of continuity. Secondly, the results indicated an increased discrepancy in adults between the mean scores in (verbal and non-verbal) reasoning vs. processing (working memory and speed) subtests. The sole exception was the Coding subtest, in which adults and children encountered similar difficulty. The reason behind it is probably linked to the fact that this subtest requires simple competencies, like managing digits and handwriting, which have been automatized by adults with SLDs. Nonetheless, the amplification of the disparity between reasoning and processing abilities in adults could be attributed to these weaknesses being deeply ingrained in individuals with SLDs. Such deep-rooted challenges might render them less adaptable to compensation through other abilities. Alternatively, the severe weaknesses observed in our sample could stem from their representation of a subgroup within the SLD population who met particular difficulties in everyday life. Only a longitudinal study comparing the same individuals at different ages would provide a clearer understanding of cognitive changes over time. Gunnel Ingesson (2006) did so and found a pattern different from that observed in the present study, with a significant decrease in verbal IQ and an increase in non-verbal IQ. The study conducted by Gunnel Ingesson (2006), however, focused solely on children during adolescence and did not track the cases as they progressed into adulthood. Furthermore, it employed an older version of the WAIS, featuring a different factorial structure. In the present study, adults with SLDs performed relatively well in the verbal reasoning subtests and were better than children in the case of the Similarity subtest. On the contrary, adults scored lower than children on two PRI subtests (Block Design and Matrix Reasoning). These results might be attributed to the fact that individuals with SLDs are still required to do inductive verbal reasoning as they age. They might be able to compensate with general abilities; meanwhile, they might not practice other forms of non-verbal reasoning, leading to diminished performance in those areas.

## 5. Conclusions

In conclusion, the present study provides crucial new insight that contributes to understanding the intellectual characteristics of adults with SLDs. Further evidence is necessary, as the study presents a series of limitations. The first limitation concerns the nature of the population assessed on a voluntary basis as its members required help for learning difficulties. Indeed, it remains unknown to what extent the participants in the study represented the whole population of adults with SLDs. The collaboration of various centers situated in different regions across Italy ensured the geographic representativeness of our sample. Additionally, the reliance on international clinical manuals for diagnosis suggests that our participants are comparable to adults diagnosed with SLDs in other parts of the world. The second limitation of the study concerns the instrument used for examining the intellectual characteristics of adults with SLDs (i.e., the WAIS-IV and its ten basic subtests). These subtests do not represent all the intellectual aspects that may be crucial in adults with SLDs. Other significant aspects, including those evaluated by the supplementary subtests (which were administered only in a limited number of cases), might also be relevant. The third limitation of the study concerns the age of the individuals, as our sample included many late adolescents and young adults (i.e., individuals who may require more assessments for learning difficulties) and fewer older adults. The correlations between age and the scores obtained on the subtests were consistently low, indicating that certain traits among individuals with SLDs stabilize significantly after the age of 16. However, we believe that a larger sample size of older adults is essential to generalize these findings across a broader age spectrum. Finally, following the suggestion of the DSM-5 (APA 2013) as well as other evidence (e.g., Cornoldi et al. 2022; Toffalini et al. 2017b), we considered the population of adults with SLDs as a whole. While it would have been interesting to explore potential distinctions among subgroups, such as those struggling with reading versus calculation, the limited number of cases with the latter profile rendered this comparison unsuitable for the present study. However, this investigation could be pursued with larger samples in the future.

Despite these limitations, we think that our study offers relevant information. At a theoretical level, it shows that the structure and characteristics of intellectual functioning may vary according to different populations, while at a practical level, it confirms the importance of conducting a multi-componential assessment of intelligence in adults with SLDs. Beyond the longstanding discussion on the approach of “strength and weaknesses” (Beaujean 2017; Toffalini et al. 2017a, 2017b), we believe that understanding the typical weaknesses in working memory and processing speed among individuals with SLDs could aid in devising strategies to mitigate the adverse effects of these limitations.

This view is coherent with a modern, non-*g*, emergent view of intelligence that argues for its use in improving equitable practices, stressing the relevance of (see also Holden and Tanenbaum 2023) lower-order specific abilities. For example, adults could become used to use clustering strategies that reduce the quantity of to-be-remembered material and have always available a tool for writing down excessive information in order to reduce memory load. Furthermore, concerning their speed limitations, they should learn to avoid strict times for meeting requests and eventually ask for a reduction of activities that require speed execution. This strategic aspect is particularly relevant for adults, for whom there is no clear evidence of the efficacy of eventual rehabilitation programs, whereas the use of responsible demanding strategies, that children do not seriously consider, might be, on the contrary, easy and motivated.

## Figures and Tables

**Table 1:** Number of cases with available data, standardized mean scores (and *SD*s), medians, and score ranges in the WAIS-IV main subtests and indexes obtained by adults with SLDs.

Measure	N	M (SD)	Median	Range (min–max)
Core Subtests				
Similarities	298	10.95 (2.95)	11	4–19
Vocabulary	298	10.19 (2.74)	10	2–19
Information	298	9.27 (3.19)	9	2–17
Block Design	298	9.69 (3.27)	10	2–19
Visual Puzzle	298	10.11 (2.89)	10	3–17
Matrix Reasoning	299	9.99 (2.88)	10	1–16
Digit Span	300	7.15 (2.93)	7	1–15
Arithmetic Reason.	296	7.34 (2.76)	7	2–15
Coding	298	8.38 (2.81)	9	1–17
Symbol Search	298	8.32 (2.86)	8	1–18
Supplementary Subtests				
Comprehension	120	10.3 (2.83)	10	3–18
Figure Weights	110	8.45 (3.24)	8	3–19
Picture Completion	116	9.2 (3.72)	9.5	1–18
Letter-Number Seq.	132	6.55 (2.39)	6	2–14
Cancellation	113	7.86 (3.38)	8	1–19
Indexes				
VCI	299	101.06 (14.44)	100	65–141
PRI	299	99.58 (15.34)	100	54–137
WMI	301	84.99 (14.19)	83	57–134
PSI	299	90.81 (13.80)	89	50–133
FSIQ	299	93.73 (13.44)	93	58–133

Note. WAIS-IV indexes, VCI = Verbal Comprehension Index; PRI = Perceptual Reasoning Index; WMI = Working Memory Index; PSI = Processing Speed Index; FSIQ = Full-Scale IQ.

**Table 2:** Mixed model parameters for both children and adults with SLD. The intercept corresponds to the estimated mean Full-Scale IQ; the other parameters reflect the estimated mean differences between each of the main indexes and the FSIQ, with 95% confidence intervals.

	Children		Adults	
Parameters	β	95% CI	β	95% CI
Intercept (FSIQ)	98.31	(97.64, 98.98)	93.76	(92.15, 95.36)
Difference VCI	4.84	(4.10, 4.59)	7.33	(5.68, 8.97)
Difference PRI	5.97	(5.23, 6.71)	5.85	(4.20, 7.49)
Difference WMI	−8.07	(−8.81, −7.33)	−8.77	(−10.42, −7.13)
Difference PSI	−5.21	(−5.95, −4.47)	−2.92	(−4.57, −1.28)

Note. Wechsler Scales Indexes, VCI = Verbal Comprehension Index; PRI = Perceptual Reasoning Index; WMI = Working Memory Index; PSI = Processing Speed Index; FSIQ = Full-Scale IQ.

## Data Availability

The data are unavailable due to privacy.

## Supplementary Materials

The following supporting information can be downloaded at: https://www.mdpi.com/article/10.3390/jintelligence11120223/s1, Table S1: Developmental sample: number of cases with available data, standardized mean scores (and SDs), medians, and scores range in the WAIS-IV main and supplementary subtests and Indexes obtained by children with SLD; Figure S1: Results of the Confirmatory Factor Analysis (standardized loadings and 95% CIs) for adults with SLD (black parameters in bold) and the population without SLD (gray parameters) based on a Four-Factor Structure, with “Arithmetic reasoning” and “Information” removed from the model; Figure S2: Results of the Confirmatory Factor Analysis (standardized loadings and 95% CIs) for adults with SLD (black parameters in bold) and the population without SLD (gray parameters) based on a Five-Factor Structure, with “Arithmetic reasoning” and “Information” removed from the model..
