# Supplementary material for: The Intellectual Profile of Adults with Specific Learning Disabilities
Source: J Intell. 2023 Dec 9;11(12):223. doi: 10.3390/jintelligence11120223 (PMC10744172; doi:10.3390/jintelligence11120223)
Supplement: Supplementary file 1 [file jintelligence-11-00223-s001.zip › jintelligence-2630086-supplementary.pdf]

Supplementary Materials

The Intellectual Profile of Adults With Specific Learning Disabilities

Table S1. Developmental sample: number of cases with available data, standardized mean scores (and SDs), medians, and scores range in the WAIS-IV main and supplementary subtests and Indexes obtained by children with SLD.

Measure	N	M (SD)	Median	Range (min – max)
**Core Subtests**				
Similarities	1610	10.26 (2.90)	10	1 – 19
Vocabulary	1610	10.38 (2.81)	10	1 – 19
Comprehension	1610	10.92 (3.20)	11	1 – 19
Block Design	1610	10.34 (2.83)	10	1 – 19
Picture Concepts	1610	11.02 (2.92)	11	1 – 19
Matrix Reasoning	1610	10.69 (3.04)	11	1 – 18
Digit Span	1610	8.17 (2.54)	8	1 – 18
Letter-Number seq.	1609	8.56 (2.55)	9	1 – 19
Coding	1610	8.36 (2.86)	8	1 – 19
Symbol Search	1610	9.31 (2.75)	9	1 – 19
**Supplementary Subtests (as available)**				
Information	495	9.41 (3.14)	9	1 – 19
Arithmetic Reasoning	590	8.46 (3.10)	9	1 – 17
Cancellation	236	9.15 (3.19)	9	1 – 18
**Indexes**				
VCI	1609	103.12 (14.80)	102	56 – 148
PRI	1609	104.27 (14.28)	104	56 – 143
WMI	1609	90.23 (12.99)	88	41 – 139
PSI	1609	93.10 (14.08)	91	47 – 144
FSIQ	1608	98.29 (12.43)	98	70 – 144

Note. WAIS-IV Indexes: VCI = Verbal Comprehension Index; PRI = Perceptual Reasoning Index; WMI = Working Memory Index; PSI = Processing Speed Index; FSIQ = Full-Scale IQ.

Figure S1. Results of the Confirmatory Factor Analysis (standardized loadings and 95% CIs) for adults with SLD (black parameters in bold) and the population without SLD (gray parameters) based on a Four-Factor Structure, with “Arithmetic reasoning” and “Information” removed from the model.

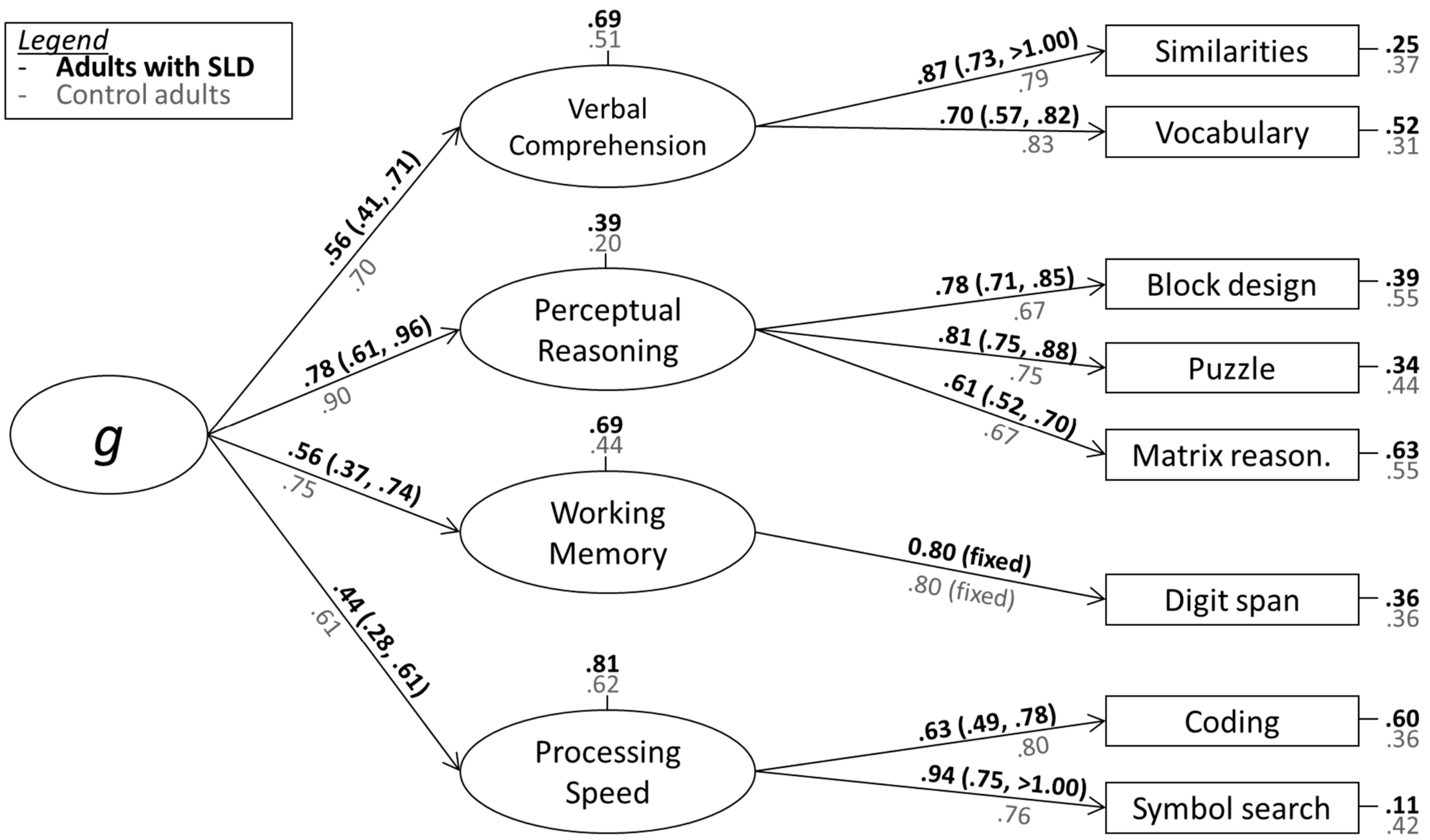

Standardized fit indices for the model on adults with SLD: $\chi 2(17) = 59.14$, $p < 0.001$, RMSEA = 0.09, SRMR = 0.05, CFI = 0.94, NNFI = 0.90.

Modification indices suggest that model could be improved with: loading of WM factor on Matrix Reasoning; direct loading of g factor on Matrix Reasoning; correlated residuals between Block Design and Puzzle (first three options).

Figure S2. Results of the Confirmatory Factor Analysis (standardized loadings and 95% CIs) for adults with SLD (black parameters in bold) and the population without SLD (gray parameters) based on a Five-Factor Structure, with "Arithmetic reasoning" and "Information" removed from the model.

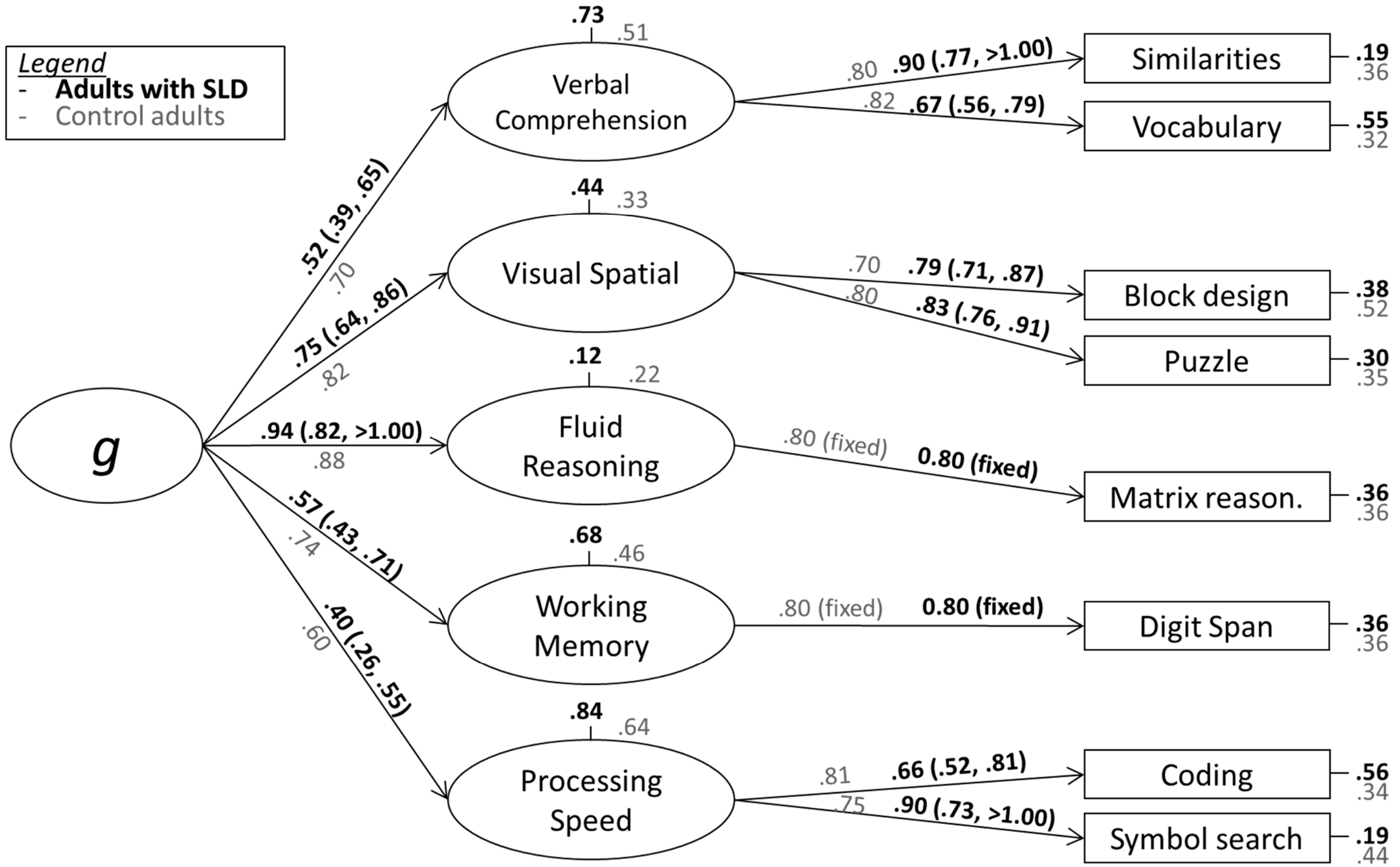

Standardized fit indices for the model on adults with SLD: $\chi 2(17) = 40.33$, $p = 0.001$, RMSEA = 0.07, SRMR = 0.04, CFI = 0.97, NNFI = 0.94
